# Implementation of the compulsory universal testing scheme in Hong Kong: Mathematical simulations of a household-based pooling approach

**DOI:** 10.3389/fpubh.2022.1053873

**Published:** 2022-12-14

**Authors:** Kei Shing Ng, Jeffrey Man Hin Hon, Stephen Chau Chun Chong, Howard Ho Kan Cheung, Jeffrey Chan, Simon Ching Lam, Benny Yiu Chung Hon

**Affiliations:** ^1^Department of Diagnostic Radiology, Li Ka Shing Faculty of Medicine, The University of Hong Kong, Pok Fu Lam, Hong Kong SAR, China; ^2^NVIDIA AI Technology Center (NVAITC), NVIDIA, Santa Clara, CA, United States; ^3^Department of Mathematics, Hong Kong Baptist University, Kowloon Tong, Hong Kong SAR, China; ^4^Department of Applied Chemistry, National Chi Nan University, Puli, Taiwan; ^5^Department of Mathematics, City University of Hong Kong, Kowloon Tong, Hong Kong SAR, China; ^6^King George V School, Ho Man Tin, Hong Kong SAR, China; ^7^School of Nursing, Tung Wah College, Ho Man Tin, Hong Kong SAR, China; ^8^Department of Psychology, University of Science and Technology of China, Hefei, China

**Keywords:** COVID-19, compulsory universal test, prevalence rate, sample pooling, mass screening, simulation

## Abstract

This study aims to propose a pooling approach to simulate the compulsory universal RT-PCR test in Hong Kong and explore the feasibility of implementing the pooling method on a household basis. The mathematical model is initially verified, and then the simulation is performed under different prevalence rates and pooled sizes. The simulated population is based in Hong Kong. The simulation included 10,000,000 swab samples, with a representative distribution of populations in Hong Kong. The samples were grouped into a batch size of 20. If the entire batch is positive, then the group is further divided into an identical group size of 10 for re-testing. Different combinations of mini-group sizes were also investigated. The proposed pooling method was extended to a household basis. A representative from each household is required to perform the RT-PCR test. Results of the simulation replications, indicate a significant reduction (*p* < 0.001) of 83.62, 64.18, and 48.46% in the testing volume for prevalence rate 1, 3, and 5%, respectively. Combined with the household-based pooling approach, the total number of RT-PCR is 437,304, 956,133, and 1,375,795 for prevalence rates 1, 3, and 5%, respectively. The household-based pooling strategy showed efficiency when the prevalence rates in the population were low. This pooling strategy can rapidly screen people in high-risk groups for COVID-19 infections and quarantine those who test positive, even when time and resources for testing are limited.

## 1. Introduction

**Figure 1:**
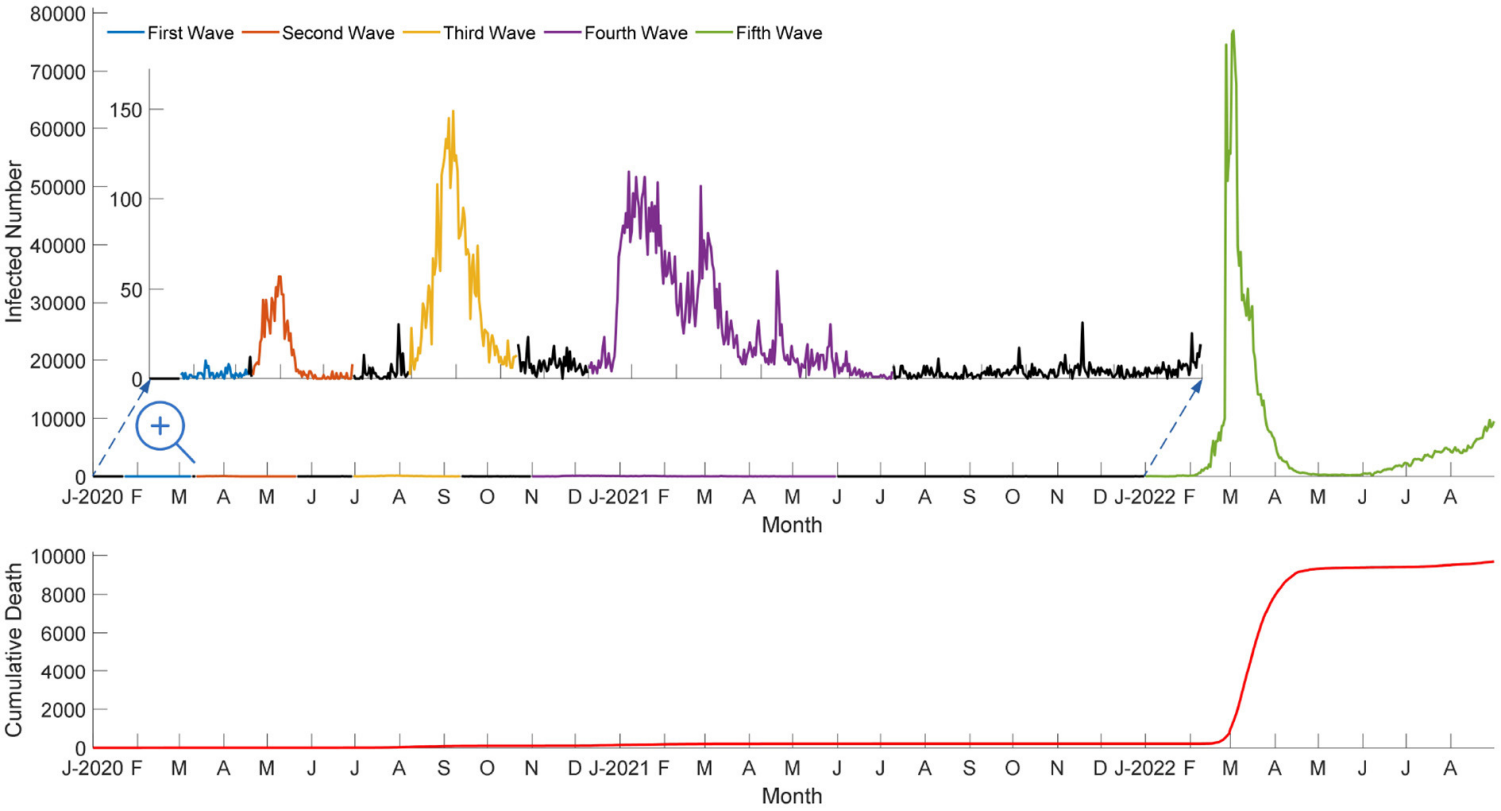
Number of infected and death cases during the multiple waves in Hong Kong from 2020 to 2022.

The fifth wave of the COVID-19 outbreak in Hong Kong is ferocious (1–3). In March 2022, Hong Kong had a chaotic epidemic period caused by the highly transmissible Omicron variant strain (4). Until August 31, 2022, over 1,530,000 cases tested positive for COVID-19, of which over 9,600 deaths were recorded. As a result, the Hong Kong government considered universal COVID-19 screening to combat the explosive fifth wave. Figure 1 shows the number of infected and death cases during the multiple waves in Hong Kong from January 2020 to August 2022.

**Figure 2:**
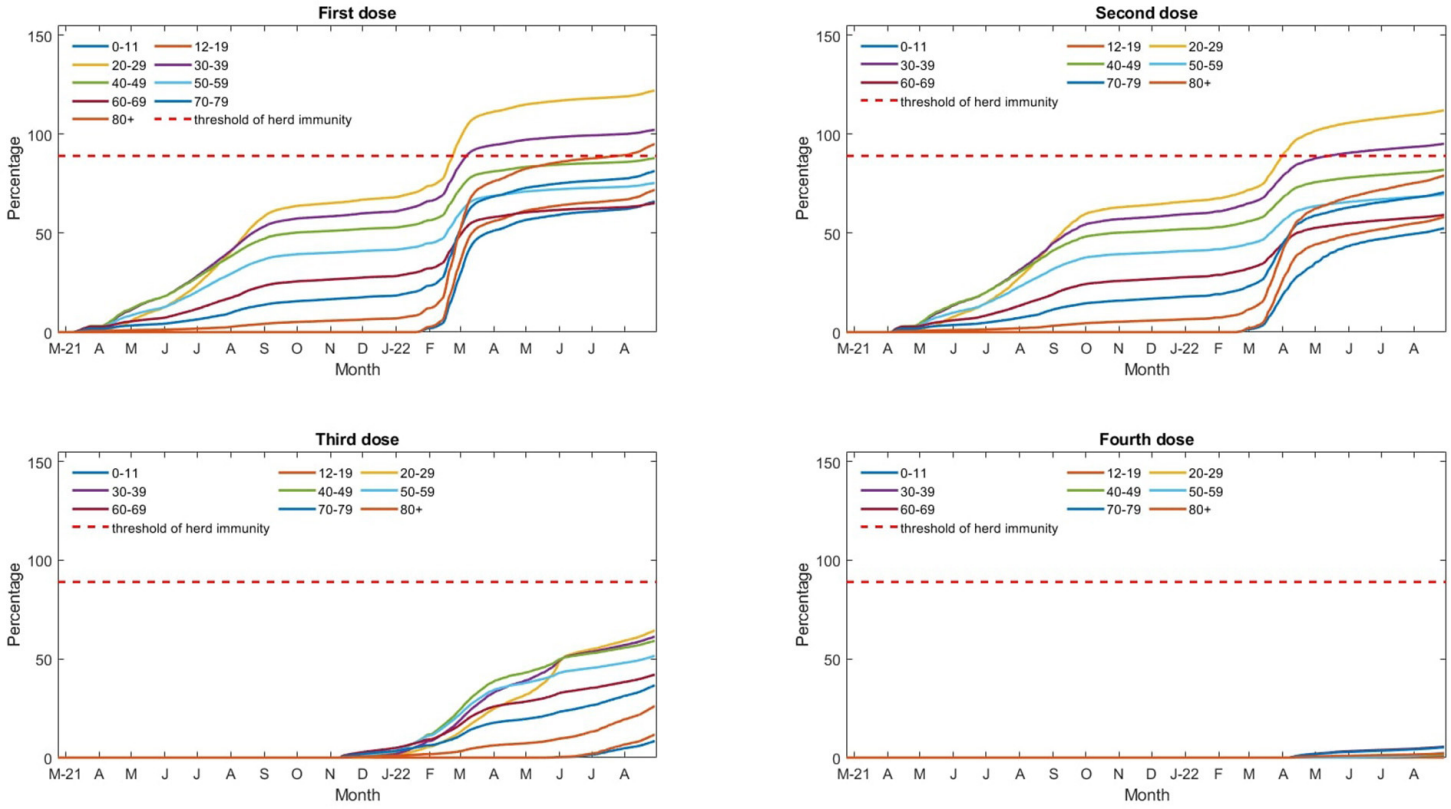
Vaccination rate for different age groups since 22 February 2021. The vaccination rate includes non-Hong Kong residents, such as holders of the Exit–Entry Permit for Traveling to and from Hong Kong and Macao with complete two doses of vaccination within their stay limit without extension, non-refoulement claimants and refugees, and other visitors staying in Hong Kong. The vaccination rate may be greater than 100% for some age groups.

Hong Kong's plan to test the entire population for coronavirus in March has been postponed indefinitely because the city prioritizes vaccinations for the elderly (5), as shown in Figure 2.

In addition, the Secretary of Health of the Government of Hong Kong, confirmed that the country would continue to adhere to China's Zero-COVID policy (6, 7). The government may reactivate the compulsory universal test (CUT) to estimate the infectious rate at a certain strategic time-point prior to achieving herd immunity (8, 9). However, no effective and viable method is available to perform population-based CUT within a short period of time due to Hong Kong's weak capacity for community organizations. Several countries have implemented mass screening strategies to slow the spread of the virus and reduce lockdown restrictions (10, 11). This study aims to develop a simulation model of a pooled testing strategy by combining subsamples to test them in groups. This pooling method (12–16) is more cost-effective for population-based testing. The average cost per individual tested in China is approximately 9 RMB Yuan (US$ 1.50) using a 10:1 pooled sampling strategy (17, 18). The household-based pooling methods do not require the entire city's lockdown and affect the citizen's daily activities. More importantly, the total number of RT-PCR tests based on a household-based pooling scheme is manageable by Hong Kong's laboratories.

## 2. Methods

### 2.1. Pooling method: An introduction

A sample of 100 individuals, in which each person has an equal chance of *r*, is considered already infected. The conventional approach requires 100 tests to determine whether a sample is positive. We combine the individual samples into five batches of 20 samples. If any one batch tested positive, then every sample inside the batch would be tested individually, thereby requiring a total of 1+20 = 21 tests. The probability of more than one positive sample occurring in a single batch is (1−(1−*r*)^20^); thus, the expected number of tests with a pooled size of 20 is as follows:


(1) $$\begin{array}{l} \text{𝔼}\left[\text{Number of tests}\right]=5+100\times \left(1-{\left(1-r\right)}^{20}\right). \end{array}$$

**Figure 3:**
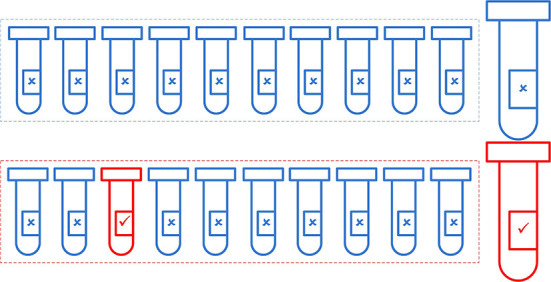
Illustration of the use of the pooling method to identify all the infected individuals in a group of 20, using pools with a size of 10. Assuming that one individual is infected (*r* = 0.05), 12 (two pooled tests + 10 individual tests) tests are required to identify all individuals.

For example, if *r* = 0.01, then the expected number of tests will be 23.2. Figure 3 illustrates the efficiency of using the pooling method of 20 individuals combined into two batches of 10 samples.

**Figure 4:**
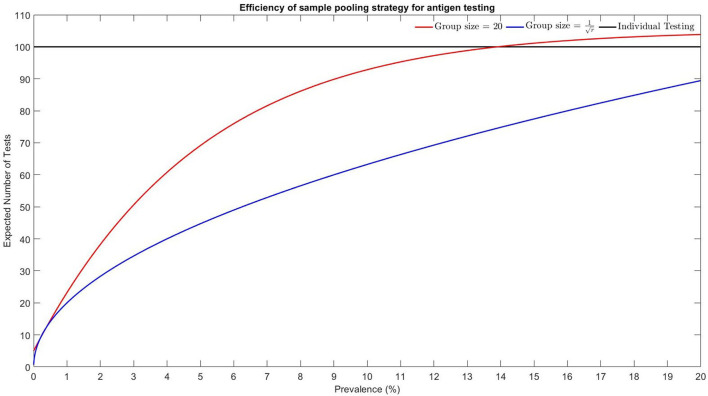
Efficiency of sample pooling strategy for antigen testing using different batch sizes.

The Dorfman's algorithm (19) suggests that the batch size should be equal to the following:


(2) $$\begin{array}{l} G=\frac{1}{\sqrt{r}} \end{array}$$

and the expected number of tests for 100 individuals is approximately equal to the following:


(3) $$\begin{array}{l} \text{𝔼}\left[\text{Number of tests}\right]\approx 2\times \sqrt{r}\times 100, \end{array}$$

when *r* is small. Figure 4 shows the efficiency of using a batch size of *G* = 20 and $G=\frac{1}{\sqrt{r}}$ and the expected number of tests for 100 individuals.

**Figure 5:**
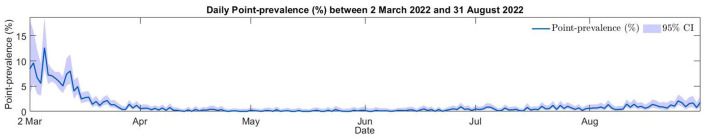
Daily prevalence rate in Hong Kong and its 95% confidence interval, adopted from https://covid19.sph.hku.hk/dashboard.

Dorfman's algorithm outperformed the pooling method with a group size of 20. For a low prevalence rate, a group size of 20 is suggested to be adopted in laboratory settings. For prevalence rates over 13.9%, the pooling method has no advantages over individual testing. Figure 5 shows the prevalence rate in Hong Kong between 2 March 2022 and 31 August 2022. Although the daily point-prevalence rate in early March was high, the implementation of antigen test for the entire population was not feasible.

### 2.2. Pooling method: Re-test with mini-pool for batches with positive results

First, 100 samples are grouped with the pool size = 20. If the entire batch is negative, then all individual samples from the batch are negative. Otherwise, the batch should be further divided into two identical mini-pools each with pool size = 10. The expected number of tests with an initial pool size of 20 and subsequent mini-pool size of 10 is obtained as follows:


(4) $$\begin{array}{l} \text{𝔼}\left[\text{Number of tests}\right]=5+10\times \left(1-{\left(1-r\right)}^{20}\right)+100\times \left(1-{\left(1-r\right)}^{10}\right). \end{array}$$

**Figure 6:**
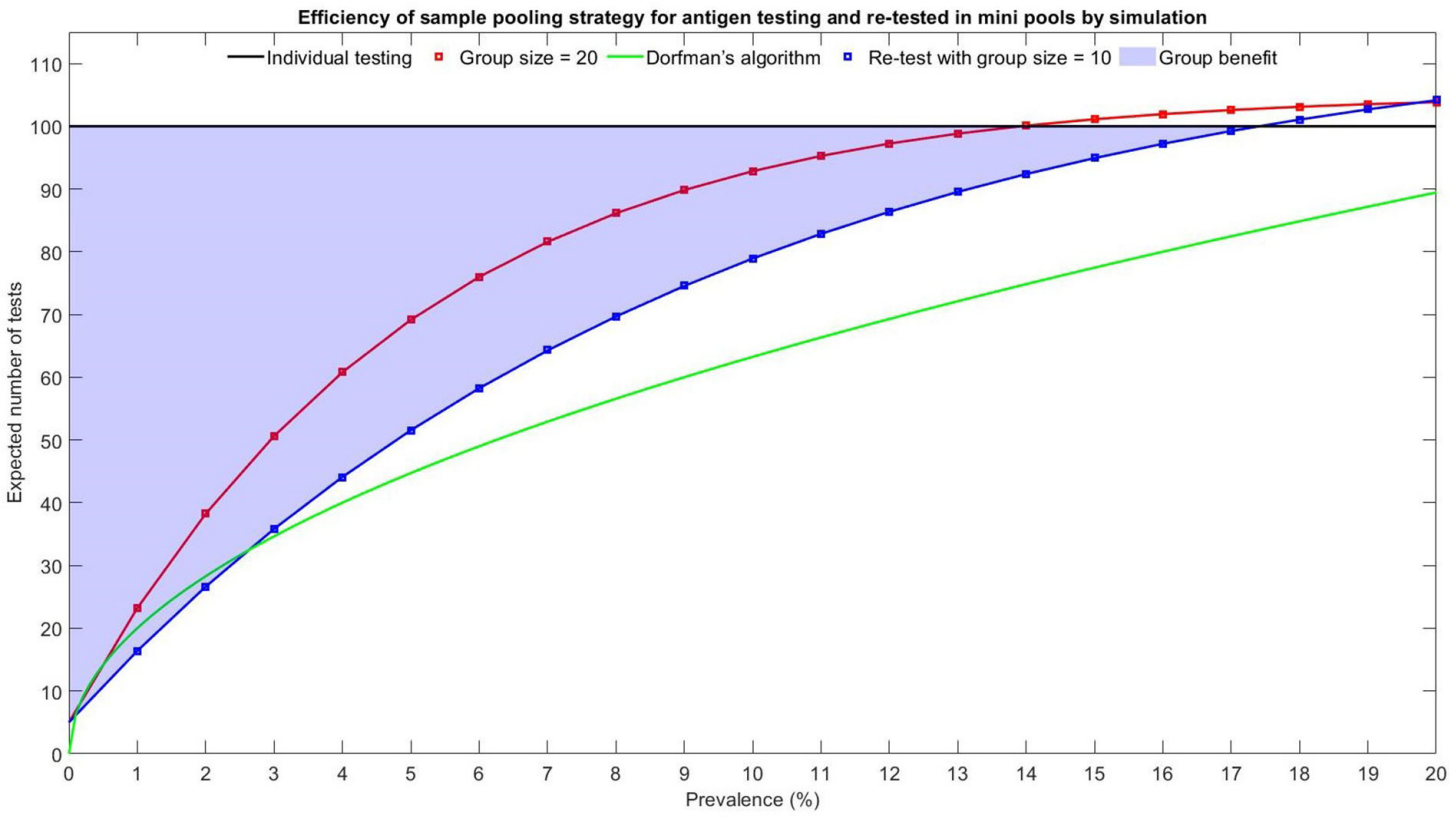
Efficiency of sample pooling strategy for antigen re-testing using mini-pools of size 10.

If *r* = 0.01, then the expected number of tests is 16.4, saving 29% of testing volumes. Figure 6 shows the Monte Carlo simulation method (20) for pooling with group size = 20 and re-test with mini-pools of size = 10. The shaded region indicates that the group benefits are higher for a lower prevalence rate. Dorfman's algorithm has a more significant group benefit for prevalence rates over 2.60%, and the pooling method with re-test using mini-pools of size = 10 is not recommended if *r*>0.174. Table 1 shows the comparison of the efficiency of different pooling strategies.

**Table 1:** Efficiency of different pooling strategies and group benefits for 100 individuals.

**Prevalence rate**	**GS = 20**	**Group benefit**	**Dorfman's algorithm**	**Group benefit**	**GS = 20, retest GS = 10**	**Group benefit**
1	23.21	76.79	20.00	80.00	16.38	83.62
2	38.24	61.76	28.28	71.72	26.62	73.38
3	50.62	49.38	34.64	65.36	35.82	64.18
4	60.80	39.20	40.00	60.00	44.10	55.90
5	69.15	30.85	44.72	55.28	51.54	48.46
6	75.99	24.01	48.99	51.01	58.24	41.76
7	81.58	18.42	52.92	47.08	64.26	35.74
8	86.13	13.87	56.57	43.43	69.67	30.33
9	89.84	10.16	60.00	40.00	74.54	25.46
10	92.84	7.16	63.25	36.75	78.92	21.08

Different combinations of mini-pool sizes have been included for comparison. Equations (5–7) show the expected number of tests with an initial pool size of 20, and subsequent mini-pool sizes of 5, 4, and 2 are given by the following:


(5) $$\begin{array}{l} \text{𝔼}[\text{Number of tests with mini-pool size = 5}]\\ =5+20\times (1-(1-r{)}^{20})+100\times (1-(1-r{)}^{5}). \end{array}$$

(6) $$\begin{array}{l} \text{𝔼}[\text{Number of tests with mini-pool size = 4}]\\ =5+25\times (1-(1-r{)}^{20})+100\times (1-(1-r{)}^{4}). \end{array}$$

(7) $$\begin{array}{l} \text{𝔼}[\text{Number of tests with mini-pool size = 2}]\\ =5+50\times (1-(1-r{)}^{20})+100\times (1-(1-r{)}^{2}). \end{array}$$

Table 2 shows the expected number of test required for different mini-pool sizes.

**Table 2:** Expected number of tests using different mini-pool sizes.

**Prevalence rate**	**Group size = 20**	**Mini-pool = 10**	**Mini-pool = 5**	**Mini-pool = 4**	**Mini-pool = 2**
1	23.21	16.38	13.54	13.49	16.09
2	38.24	26.62	21.26	21.07	25.58
3	50.62	35.82	28.25	27.88	33.72
4	60.80	44.10	34.62	34.02	40.74
5	69.15	51.54	40.45	39.59	46.83
6	75.99	58.24	45.81	44.67	52.13
7	81.58	64.26	50.75	49.34	56.80
8	86.13	69.67	55.32	53.64	60.93
9	89.84	74.54	59.56	57.63	64.61
10	92.84	78.92	63.52	61.35	67.92

Table 2 shows that, for a low prevalence rate, a re-test with a mini-pool size = 2 is not a better option than the mini-pool size of 10. Although re-testing with a mini-pool size equal to 4 or 5 further reduces the expected number of tests significantly, the mini-pool size of 10 is still recommended. The reason for not selecting a smaller mini-pool size for follow-up testing is to avoid creating too many subgroups, which require additional time and storage space in the laboratory. For high quantities of samples, subdividing them into multiples of 10 is more manageable than grouping them in a batch of 4 or 5. Therefore, a trade-off between the pooling method and the turnaround time of the testing results is necessary. A *p* < 0.05 was considered statistically significant.

## 3. Results and discussion

### 3.1. Implementation of household-based pooling method in Hong Kong

**Figure 7:**
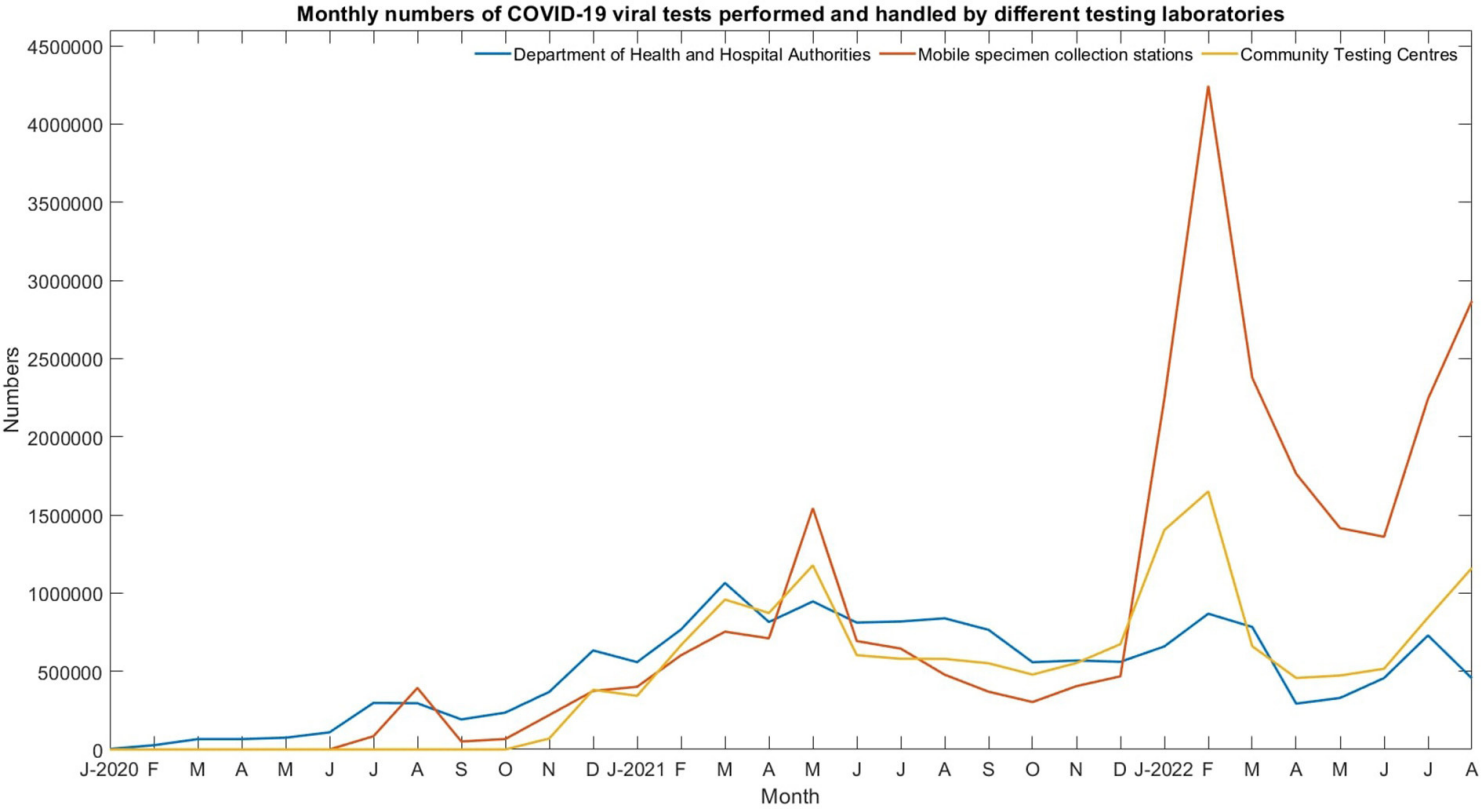
Monthly numbers of COVID-19 viral tests performed and handled by the Department of Health, Hospital Authority, mobile specimen collection stations, and the Community Testing Centers.

**Figure 8:**
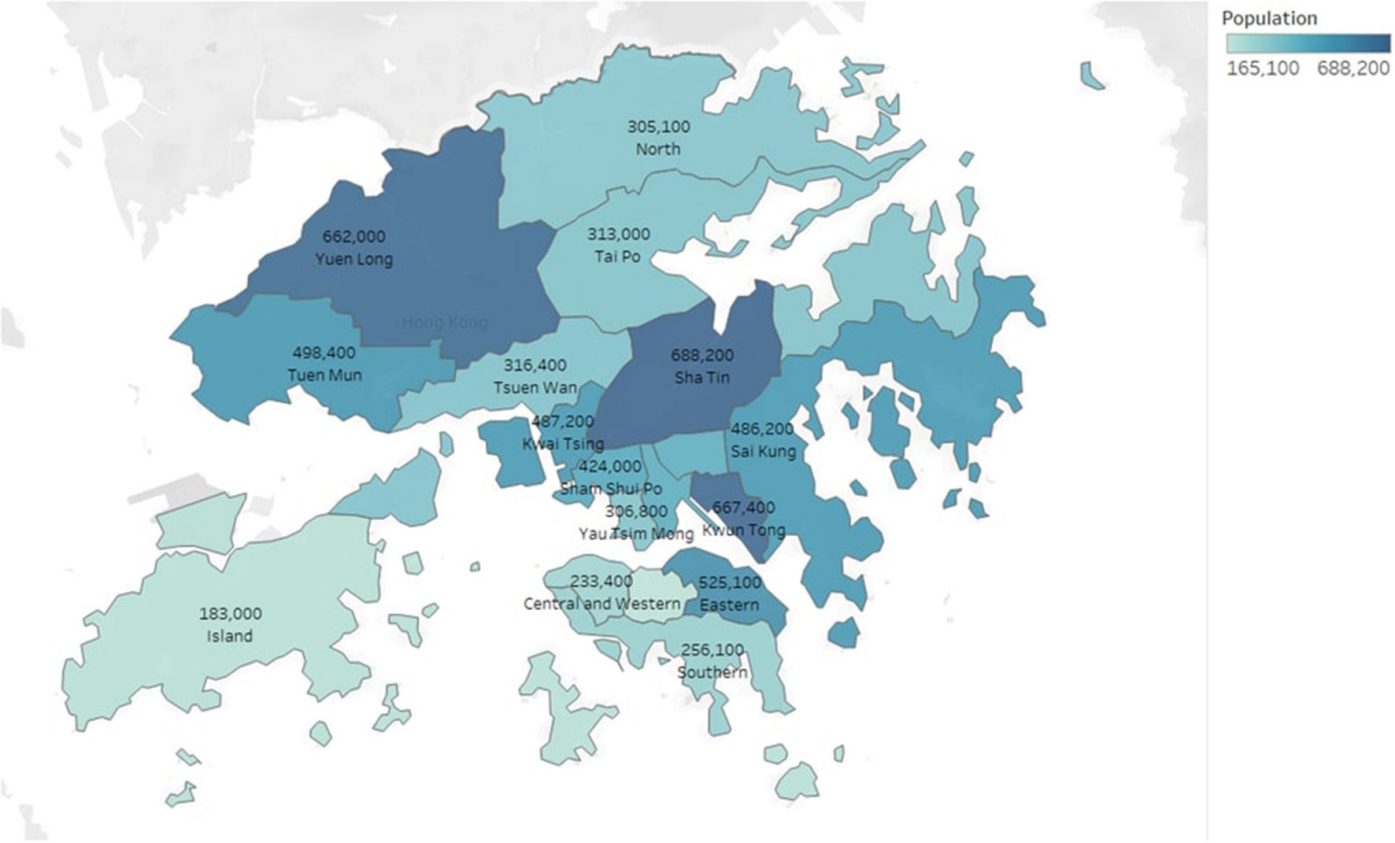
Distribution of populations in Hong Kong.

Universal testing is an effective tool for eliminating silent transmissions. It allows Hong Kong to resume social and economic activities while providing a foundation for the city to argue for the reinstatement of quarantine-free travel with the mainland. According to the Census and Statistics Department, Hong Kong's population was 7,324,600 in 2021. However, at this stage of the epidemic, population-based universal testing would be costly and ineffective because it would requires multiple rounds of testing and the isolation of a significant number of non-infectious persons. Statistics shows that the maximum average daily number of COVID-19 viral tests performed and handled by the Department of Health, Hospital Authority, mobile specimen collection stations and the Community Testing Centers was around 241,000 in February, 2022. Figure 7 shows the monthly numbers of COVID-19 viral tests performed and handled by different testing laboratories. According to the most optimistic forecasts, this universal test requires at least 1–2 weeks to reach the goal for population-based testing. Studies showed that testing the population by sampling can reduce the spread of the disease (21). A household-based COVID antigen test is proposed to further reduce the large number of individual tests required for a densely populated city like Hong Kong. Hong Kong has approximately 2.67 million households with family sizes ranging from one to six or more members (Table 3). Figure 8 shows the population distribution in Hong Kong.

**Table 3:** Household sizes and numbers.

**Household sizes**	Number^*^
1	522,900
2	785,300
3	636,100
4	469,100
5	178,500
6 or more	77,400
Total	2,669,300

* Provisional figures in 2021.

Hong Kong has a high population density, ranking fourth in the world, with approxmiately 7,060 people per km^2^ in 2021. Thus, controlling respiratory infections is challenging (22). An Existing quarantine rule for the patient with COVID-19 is that if a COVID patient's family only has one toilet, then the Center for Health Protection will arrange for all family members to be quarantined in designated facilities. A family usually has only one toilet in Hong Kong; thus, we propose that each household assigns a family member to perform the antigen test within the testing period. Table 4 shows the distribution of the family sizes across the territories of Hong Kong and the number of tests required under prevalence rates of *r* = 0.01, 0.03, and 0.05. If the test result from a family member is positive, then the rest of the family members must also undergo the compulsory antigen test because they were considered as ‘close contacts' of COVID-19 patients.

**Table 4:** Number of tests required for different districts and the prevalence rate in Hong Kong.

**District**	Population^*^	# of Household^*^	**# of Tests**		
			*r* **= 0.01**	*r* **= 0.03**	*r* **= 0.05**
Wan Chai	165,100	63,200	10,353.88	22,638.01	32,574.19
Island	183,000	67,400	11,041.96	24,142.44	34,738.94
Central and Western	233,400	87,700	14,367.65	31,413.83	45,201.85
Southern	256,100	86,300	14,138.29	30,912.35	44,480.27
North	305,100	111,300	18,233.97	39,867.26	57,365.63
Yau Tsim Mong	306,800	123,600	20,249.05	44,273.08	63,705.23
Tai Po	313,000	110,600	18,119.29	39,616.53	57,004.84
Tsuen Wan	316,400	114,000	18,676.30	40,834.39	58,757.25
Wong Tai Sin	403,000	147,200	24,115.37	52,726.52	75,869.01
Kowloon City	404,200	146,500	24,000.69	52,475.78	75,508.22
Sham Shui Po	424,000	164,700	26,982.34	58,994.95	84,888.76
Sai Kung	486,200	166,800	27,326.38	59,747.17	85,971.13
Kwai Tsing	487,200	176,600	28,931.89	63,257.49	91,022.20
Tuen Mun	498,400	187,200	30,668.46	67,054.37	96,485.59
Eastern	525,100	188,400	30,865.05	67,484.21	97,104.09
Yuen Long	662,000	234,700	38,450.25	84,068.70	120,967.80
Kwun Tong	667,400	247,400	40,530.86	88,617.80	127,513.50
Sha Tin	688,200	245,700	40,252.35	88,008.87	126,637.33
Whole Territory	7,324,600	2,669,300	437,304.03	956,133.75	1,375,795.84

* Provisional figures in 2021.

Hong Kong will make COVID RT-PCR tests available within walking distance for approximately five million population in the late October 2022. In addition, Hong Kong will transform part of its mobile stations into community testing centers, creating a network of 85 facilities. Thus, approximately 70% of the population can access the community testing centers within a 15-min walk. The maximum number of COVID PCR tests performed was approximately 0.25 million per day in February 2022.

We developed three recommendations to implement the CUT to serve 2.67 million households within 2 days with limited resources. First, the number of testing vehicles should be increased so that the specimen collection centers can cover at least 90% of the population within a 15-min walk. Second, 500% more temporary staffs, such as swabbers and testing technicians, should be recruited. The additional workforce can be achieved by recruiting undergraduate students majoring in Nursing, Medical laboratory science, Medicine, and nurses from community vaccination centers. Third, the laboratory capability, such as setting up an air-inflated laboratory in the local sports center and utilizing local universities' laboratory resources, should be increased.

The CUT requires participants to register and take the swabs for only a few minutes. In addition, the Hong Kong government has experience in holding territory-wide events. For example, over 4.1 million eligible citizens in Hong Kong casted their votes in the 2019 district council elections in 15 hours.

As shown in Table 4, 63.56% reduction in testing the number of swab samples was observed, indicating a decrease from 7,324,600 (population-based) to 2,669,300 (household-based), which should be collected for the population-household-based studies. The number of tests will be reduced to 437,304, using the pooling test and re-test strategy under a 1% prevalence rate; only 5.97% of the testing resources is required in Hong Kong's laboratories.

### 3.2. Implications to the other countries

Providing test results rapidly to cut off transmission chains is critical. Since the onset of the pandemic, Macau has only reported six deaths and 2,344 infections (793 confirmed cases and 1,551 asymptomatic). Similar to the rest of China, the region has adopted the dynamic Zero-COVID strategy, which relies on population mass testing, isolation, and lockdowns to reduce the spread of the coronavirus. As a result, the Macau government allows citizens to travel to Zhuhai, China, without quarantine. In addition, quarantine-free travel between mainland China and Hong Kong has been suspended since 2020. The universal test can help the Hong Kong government better understand the current pandemic situation, cut the transmission chains, and connect with the rest of the world by relaxing travel restrictions. Hong Kong can attract bankers, fund managers, and other finance professionals to return to the city. In addition, fully reopening the border between Hong Kong and China can alleviate the poor economic situation in Hong Kong and the mainland. Hong Kong is China's gateway to the global financial system, a crucial conduit to the west for foreign capital and trade. Up to 10 October 2022, less than 20 countries still have quarantine requirements for inbound travelers. For example, even fully vaccinated travelers from the United States will be denied entry into China for tourism purposes. Therefore, mass antigen testing might aid in mitigating the epidemic and re-opening the economy. We recommend that other countries can implement multiple rounds of the universal test using the pooling method, two rounds of the antigen test could reduce the spread of diseases by 80% (11).

### 3.3. Limitations

The cycle threshold (CT) values of positive specimens from patients at the time of diagnosis were not considered a limitation of pooling. However, samples with CT values greater than 30, indicating low virus titer, are expected to be vulnerable to pooling. The distribution of CT values in the actual population should be considered when determining pool size. The clinical sensitivity of pooled specimens at a given pool size is significant because the pooled samples are expected to be used as a screening tool. To get a higher sensitivity of the pooling method, the grouping of the PCR samples could be according to the participants' risk factors. For example, people who have received three or more doses of vaccination or with a history of infection should be considered the low-risk group. Conversely, those who received one dose or less should be regarded as the high-risk group; their CT value will be lower if infected.

## 4. Conclusions

After analyzing scientific evidence and establishing a balance among factors, such as transmission risks, the Hong Kong government announced that the mandatory quarantine rule for arriving individuals from outside Hong Kong would be lifted beginning September 26, 2022. Hong Kong has introduced a “0+3” period for visitors, indicating no compulsory quarantine is required, instead they should self-monitor their symptoms for 3 days. The government's objective is to minimize the hardship caused by quarantine procedures for coming travelers while allowing Hong Kong to interact with the rest of the world as much as possible and controlling the spread of the epidemic. The Hong Kong government should re-consider the compulsory universal COVID-19 screening to estimate the current infectious rate and take the preventive measures to combat the potential outbreak of the sixth wave.

The proposed household-based pooling strategy showed efficiency when the prevalence rates in the population were low. This pooling strategy can rapidly screen people in high-risk groups for COVID-19 infections and quarantine those who test positive, even when time and resources for testing are limited. Symptomatic patients should be tested individually rather than in groups.

Different from mainland China, Hong Kong's weak capacity for community organizations causes difficulty in conducting population-based testing. Therefore, the household-based pooling approach can play an integral role in controlling the spread of COVID-19 effectively.

## Data availability statement

The raw data supporting the conclusions of this article will be made available by the authors, without undue reservation.

## Author contributions

BH, KN, and SL: conception and design. JH, BH, SL, and KN: data analysis and interpretation. BH and SL: administrative support, full access to all the data in the study, and had final responsibility for the decision to submit for publication. All authors wrote the manuscript and approved the final of manuscript.
